# Eukaryotic translation initiation factor 4AII contributes to microRNA-122 regulation of hepatitis C virus replication

**DOI:** 10.1093/nar/gky262

**Published:** 2018-04-14

**Authors:** Choudhary Shoaib Ahmed, Poppy L Winlow, Aimee L Parsons, Catherine L Jopling

**Affiliations:** School of Pharmacy, University of Nottingham, University Park, Nottingham NG7 2RD, UK

## Abstract

Hepatitis C virus (HCV) is a positive sense RNA virus that persistently infects human liver, leading to cirrhosis and hepatocellular carcinoma. HCV replication requires the liver-specific microRNA-122 (miR-122). In contrast to canonical miRNA-mediated repression via 3′UTR sites, miR-122 positively regulates HCV replication by a direct interaction with the 5′ untranslated region (UTR) of the viral RNA. The protein factor requirements for this unusual miRNA regulation remain poorly understood. Here, we identify eIF4AII, previously implicated in miRNA-mediated repression via 3′UTR sites, as a host factor that is important for HCV replication. We demonstrate that eIF4AII interacts with HCV RNA and that this interaction is miR-122-dependent. We show that effective miR-122 binding to, and regulation of, HCV RNA are reduced following eIF4AII depletion. We find that the previously identified HCV co-factor CNOT1, which has also been implicated in miRNA-mediated repression via 3′UTR sites, contributes to regulation of HCV by eIF4AII. Finally, we show that eIF4AI knockdown alleviates the inhibition of HCV replication mediated by depletion of either eIF4AII or CNOT1. Our results suggest a competition effect between the eIF4A proteins to influence HCV replication by modulation of miR-122 function.

## INTRODUCTION

HCV is a major cause of chronic liver disease worldwide, infecting >184 million people (1). While the recent development of direct acting antiviral drugs shows great promise in HCV therapy, issues such as the high cost of these drugs make it important that research into the HCV life cycle continues (2). Replication of the 9.6 kilobase (kb) HCV RNA begins with translation of the viral polyprotein, driven by an internal ribosome entry site (IRES) that directly recruits the 40S ribosomal subunit and eukaryotic initiation factor (eIF)3. Importantly, the HCV IRES does not require any components of the cap binding complex, including eIF4A (3). The viral polyprotein is cleaved by host and viral proteases to generate HCV structural and nonstructural proteins. The viral non-structural proteins are essential for RNA replication, which occurs in an endoplasmic reticulum-derived membranous web. The RNA-dependent RNA polymerase NS5B mediates negative and positive sense HCV RNA synthesis (4).

The liver-specific microRNA-122 (miR-122) is essential for HCV replication (5). miRNAs are 21–23 nucleotide (nt) single-stranded RNA molecules expressed by most eukaryotic organisms that generally function in animals by interacting with partially complementary sites in the 3′ untranslated region (UTR) of mRNA targets, leading to translation inhibition and mRNA degradation. miRNAs bind to targets in association with a complex of proteins known as the RNAi-induced silencing complex (RISC), in which an Argonaute protein (Ago1–4 in mammals) is the central component (6). In contrast, miR-122 binds to two adjacent sites in the 5′UTR of HCV RNA, upstream of the IRES, and positively regulates viral replication (5,7). The mechanism of regulation has been difficult to elucidate. Enhancement of HCV IRES-driven translation by miR-122 binding was observed in some studies (8–10). However, this was not seen in a number of other studies, which instead found that miR-122 binding to the HCV 5′UTR masks the 5′ nucleotides of HCV and protects HCV RNA from degradation mediated by the 5′-3′ exonucleases Xrn1 (11,12) or Xrn2 (13). Importantly, translation regulation and/or RNA stabilization are not sufficient to explain the full effect of miR-122 on the HCV replication cycle (11,14). A recent explanation for the missing element of miR-122 regulation was found when miR-122 was shown to promote the switch from translation to RNA replication by displacing poly(rC)-binding protein 2 (PCBP2) from the HCV 5′UTR (15). However, many unanswered questions regarding the mechanism of miR-122 regulation of HCV remain, including whether miR-122 functions in association with a canonical RISC and whether other protein factors can modulate its activity.

miRNA-mediated repression of translation by binding to 3′UTR sites was shown to require the DEAD-box RNA helicase eIF4AII (16). eIF4AII is a close homologue of eIF4AI, and has generally been thought to function in an identical fashion by unwinding 5′UTR secondary structure within the cap-binding eIF4F complex, promoting ribosomal scanning and translation initiation. However, eIF4AII is not able to functionally replace eIF4AI (17), implying that the two proteins have different functions. A yeast two hybrid screen for proteins that interact directly with HCV NS5B identified eIF4AII but not eIF4AI (18). Based on this observation and the role for eIF4AII in miRNA function, we investigated whether eIF4AII contributes to HCV replication and its regulation by miR-122.

Here, we show that eIF4AII is required for efficient HCV replication in several different systems. eIF4AII interacts with HCV replicon RNA and this interaction requires miR-122. We find that eIF4AII contributes to HCV IRES-driven translation and to its regulation by miR-122. By immunoprecipitation, we observe a decrease in association of Argonaute with HCV RNA under conditions of eIF4AII depletion, suggesting that eIF4AII is required for efficient miR-122 recruitment to, or retention on, the viral RNA. We also investigate the protein CNOT1, which is a host factor for HCV and also required for miRNA-mediated repression via 3′UTR sites, and find that it contributes to the eIF4AII–HCV RNA interaction. Finally, we show that eIF4AI depletion relieves the inhibition of HCV replication by eIF4AII or CNOT1 knockdown, suggesting eIF4AII and CNOT1 are only required for HCV replication when eIF4AI is present. Taken together, our results identify eIF4AII as a new host factor that is important for HCV replication, describe a novel and unexpected role for part of the eukaryotic translation initiation machinery, and provide insight into the little-understood role for miR-122 in HCV replication.

## MATERIALS AND METHODS

### Plasmids, *in vitro* transcription and RNA oligonucleotides

The plasmid p5′LUC3′ has been described previously (9). pH77ΔE1/p7 was a kind gift of Stanley Lemon (19). Two plasmids encoding infectious HCV RNAs, pBi-Gluc-H77C(1a)/JFH and pFL-J6/JFH1, were kind gifts of Charles Rice (20). *In vitro* transcription was carried out using the T7 Megascript kit (Ambion) according to the manufacturer's instructions, with *EcoRI*-linearized p5′LUC3′ or *XbaI*-linearized pH77ΔE1/p7, pBi-Gluc-H77C(1a)/JFH1 or pFL-J6/JFH1 as templates. The capped, polyadenylated *Renilla* luciferase transfection control RNA was synthesized from a linearized pSV40-RL (Promega) template using the mMessage mMachine kit (Ambion) and polyadenylated using the Poly(A) tailing kit (Ambion). The miRNA duplexes miR122wt and miR122p3+4, and the 2′-O-methylated oligonucleotides Rand-2’OMe and 122-2’OMe (referred to here as control oligo and anti-miR-122, respectively), have been described previously (9).

### Cell culture, transfection and viral infection

Huh7 and Huh7.5 cells were cultured as previously described (9). Ambion silencer select siRNAs used were eIF4AI si (s4567), eIF4AII si1 (s4572), eIF4AII si2 (s4570) and CNOT1 si (s22844). Control si1 was an ON-TargetPLUS non-targeting siRNA #3 purchased from Dharmacon, and control si2 an Ambion silencer select non-targeting siRNA (AM4613). All were delivered into cells at 10 nM final concentration using Lipofectamine RNAiMax (Invitrogen). Cells were cultured for 72 h before harvesting. For luciferase experiments, cells were transfected with siRNAs in 6 cm plates and cultured for 48 h before splitting into 24-well plates. Transfection of 5′LUC3′ RNA, *Renilla* luciferase RNA and miRNA inhibitor/duplex was carried out using lipofectamine 2000 as described previously (9). Cells were harvested at 6 h post transfection in Passive Lysis Buffer (Promega) and luciferase activity measured with the Dual luciferase assay system (Promega) using a Glomax luminometer (Promega).

Electroporation was used to introduce wildtype or mutant H77ΔE1/p7, Bi-Gluc-H77C(1a)/JFH1 or FL-J6/JFH1 RNA into Huh7 or Huh7.5 cells. Electroporation was carried out using the Neon system (Invitrogen) according to the manufacturer's instructions. Where included, siRNA treatment was for 48 h prior to electroporation. 4 × 10^5^ cells were resuspended in 10 μl buffer R and mixed with 1 μg wildtype or mutant H77ΔE1/p7, Bi-Gluc-H77C(1a)/JFH1, FL-J6/JFH1 or 5′LUC3′ RNA, and 20 pmol anti-miR-122 where included, before electroporation with a single pulse at 1300 V for 30 ms. For immunoprecipitation experiments, three electroporations were pooled and plated on a 10 cm plate. For Bi-Gluc-H77C(1a)/JFH1 RNA experiments, 10% of the electroporated cells were plated in each of three wells of a 24-well plate for luciferase assays. 10 μl of cell supernatant from triplicate wells was harvested at 1, 2, 3, 4, 6 and 24 h timepoints in luciferase lysis buffer (NEB), and assayed with *Gaussia* luciferase assay reagent (NEB).

For viral infection experiments, Huh7.5 cells were transfected with siRNAs and cultured for 24 h before J6/JFH1 virus was added at an m.o.i. of 0.01. Media was replaced 4 h after infection and total RNA was harvested at 48 h post-infection.

Silvestrol (MedChemExpress) was dissolved in DMSO and applied to cells at a final final concentration of 1 μM for 30 min. When combined with siRNA knockdown, silvestrol treatment was for 16 h.

### Immunoprecipitation and western blotting

Immunoprecipitation was performed on replicon cells, or cells electroporated with H77ΔE1/p7 RNA and cultured for 72 h, as described in (21), using Abcam polyclonal antibodies specific to eIF4AI (ab31217) or eIF4AII (ab31218) or the monoclonal antibody 2A8 (Sigma) to precipitate Ago1–4. RNA was isolated from 25% of the input cell lysate, the eIF4AI, eIF4AII or Ago1–4 immunoprecipitate, and an isotype control IgG (Santa Cruz) immunoprecipitate using TRI reagent (Sigma) and analyzed by qPCR. For western blotting, protein samples from total cell lysates were obtained by RIPA lysis. SDS-PAGE loading dye was added to total cell lysate, or for immunoprecipitation experiments to input lysate and to the immunoprecipitate, to a final concentration of 1×. Protein samples were separated by electrophoresis on 10% SDS-PAGE gels before semi-dry transfer to PVDF membrane. eIF4AI and II were detected using the antibodies given above, β-tubulin was detected using the antibody ab6046 (Abcam), and Ago2 was detected using the antibody 11A9 (Millipore).

### RNA isolation, northern blotting and quantitative RT-PCR

RNA was extracted using TRI reagent (Sigma) according to the manufacturer's protocol. Northern blot analysis of HCV and γ-actin RNA was carried out as described previously (7). Quantitative RT-PCR (qPCR) was performed using GoTaq qPCR Master Mix (Promega), following reverse transcription using Superscript III and random primers, as described in (21). qPCR to detect miR-122 and U6 snRNA was carried out using specific miRNA Taqman assay kits (Applied Biosystems) according to the manufacturer's protocol. qPCR was carried out using a Stratagene Mx3005P machine, and data were analyzed by the 2^−ΔΔCt^ method relative to the actin mRNA or U6 control for total RNA experiments, or as 2^−ΔCt^ relative to 25% input RNA for immunoprecipitation experiments.

### 4-thio-Uridine labeling

4-thio-Uridine (4SU) labeling of newly synthesised RNA was carried out in Huh7 cells electroporated with H77ΔE1/p7 RNA and plated in 10 cm plates, transfected with eIF4AII siRNA or a non-targeting control at 24 h post-electoporation, and labeled with 4SU at 48 h post-transfection. 4SU was added at a final concentration of 200 μM in fresh medium. Biotinylation and isolation of 4SU-labeled RNA was carried out as described in (22), except that MTSEA-biotin-XX (Biotium) was used as the biotinylation reagent, as described in (23). HCV and γ-actin mRNA expression in unlabeled and labeled RNA were determined by qPCR.

### Statistical analysis

All data represent mean of at least three independent biological replicates, with error bars representing standard deviation. Statistical analysis was carried out by two-tailed Student's *t* test for unpaired samples of equal variance. One sample, two-tailed *t* test was used for data compared to a normalized control value. *P < 0.05, **P < 0.01, ***P < 0.001.

## RESULTS

### eIF4AII contributes to HCV replication

**Figure 1.**
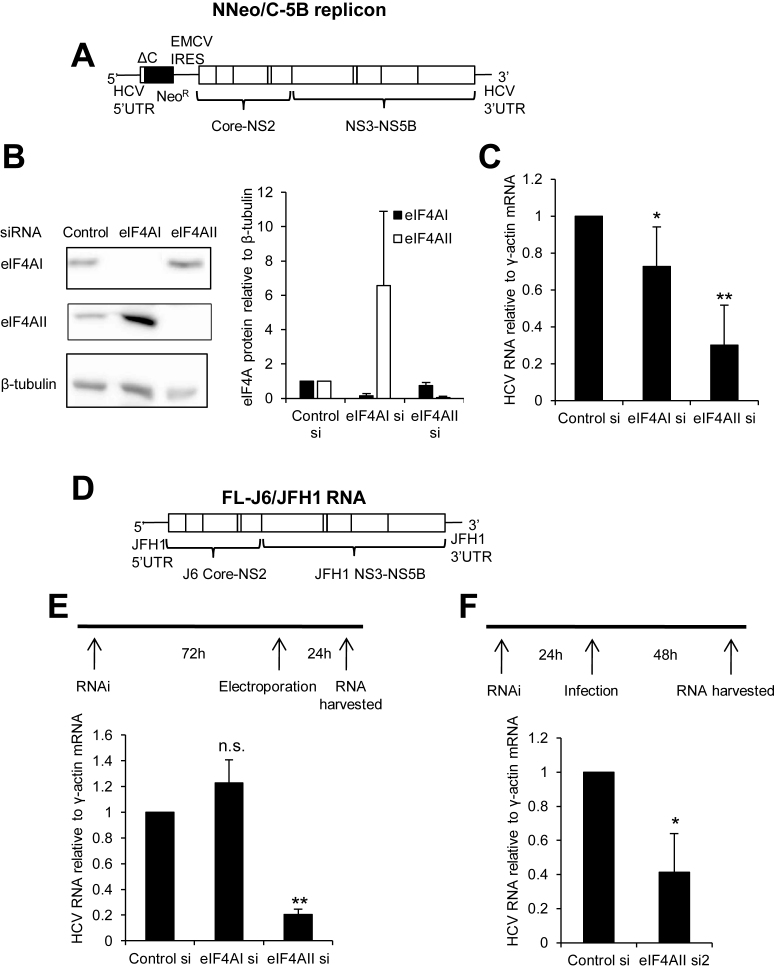
eIF4AII contributes to HCV replication. (**A**) Structure of the NNeo/C-5B bicistronic replicon RNA. (**B**) Western blot showing effective knockdown of eIF4AI and eIF4AII by respective siRNAs. β-tubulin is shown as a loading control. Graph shows quantification of eIF4AI and eIF4AII protein relative to β-tubulin, relative to control siRNA transfection. Mean of three independent experiments, +SD. (**C**) HCV RNA relative to a γ-actin mRNA control was measured by qPCR in replicon cells 72h after transfection with the indicated siRNAs. Mean of 5 independent experiments, +SD. *P = 0.048, **P = 0.0010 relative to control si, one sample Student's *t* test. (**D**) Structure of the J6/JFH1 infectious RNA. (**E**) Huh7 cells were treated with eIF4AI or eIF4AII siRNA, or a non-targeting control, for 72 h before J6/JFH1 RNA electroporation. HCV RNA relative to γ-actin mRNA was measured by qPCR at 24 h post-electroporation. Mean of 3 independent experiments, +SD. ***P = 0.0007 relative to control si, one sample Student's *t* test. (**F**) Huh7 cells were treated with eIF4AII si2, or a non-targeting control, for 24 h before infection with J6/JFH1 virus. HCV RNA relative to γ-actin mRNA was measured by qPCR at 48 h post-infection. Mean of four independent experiments, +SD. *P = 0.014 relative to control si, one sample Student's *t* test.

To determine whether eIF4AII influences HCV replication, initial experiments were carried out in Huh7 cells that stably contain a bicistronic genotype 1b HCV replicon (NNeo/C-5B) (24) (Figure 1A). siRNA transfection was used to deplete eIF4AI and eIF4AII. Depletion of both proteins was effective (Figure 1B). We also confirmed the previous observation that eIF4AII protein levels increase following eIF4AI knockdown (Figure 1B), which was shown to be due to transcriptional induction (17). eIF4AII depletion led to a 70% decrease in HCV RNA levels in the replicon cells (Figure 1C), suggesting that eIF4AII contributes to HCV replication. A smaller, but significant, decrease in HCV RNA was observed following eIF4AI knockdown. As the bicistronic HCV replicon contains the encephalomyocarditis (EMCV) IRES, which requires eIF4A for function and may influence its response to eIF4AI and II depletion, the knockdown experiments were repeated in cells electroporated with a monocistronic J6/JFH1 chimeric HCV RNA that forms infectious virus (Figure 1D). This experiment was carried out by transfecting cells with siRNAs for 72 h before the viral RNA was introduced, which allowed us to establish whether eIF4A proteins contribute to establishment of a viral replication cycle. A strong decrease in HCV RNA was observed following eIF4AII, but not eIF4AI, knockdown (Figure 1E), confirming that eIF4AII is a host factor for HCV and suggesting that the reduction in HCV RNA in replicon cells following eIF4AI knockdown is likely to be related to the presence of the EMCV IRES. To test the effect of eIF4AII on HCV replication in the context of viral infection, Huh7.5 cells were transfected with siRNA targeting eIF4AII or a non-targeting control 24 h before infection with J6/JFH1 virus. This experiment used a second siRNA to eIF4AII and confirmed that eIF4AII is a host factor for HCV replication (Figure 1F). Finally, we established that eIF4AII depletion does not affect cell proliferation, confirming the results of (17) and excluding a possible indirect effect on HCV replication due to changes in cell proliferation or viability (Supplementary Figure S1).

### eIF4AII interacts with HCV RNA and miR-122

**Figure 2.**
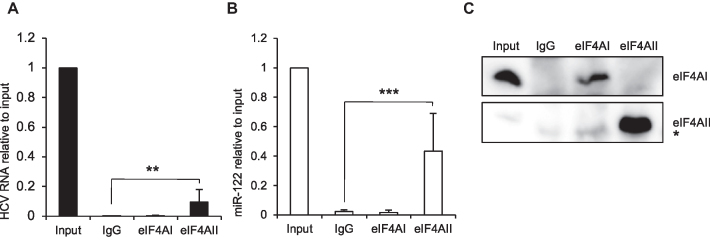
eIF4AII interacts with HCV RNA and miR-122. RNA was extracted following immunoprecipitation of Huh7 NNeo/C-5B replicon cell cytoplasmic lysates with antibodies specific to eIF4AI, eIF4AII, or an IgG control. (**A**) HCV RNA or (**B**) miR-122 in the immunoprecipitates was measured by qPCR and is shown relative to 25% input. Mean of nine independent experiments +SD. **P = 0.0089, ***P = 0.00054, Student's *t* test. (**C**) Protein was extracted from immunoprecipitates and analyzed by western blot with antibodies specific to eIF4AI or eIF4AII. 2% input was loaded. * indicates non-specific IgG band.

Having established that eIF4AII contributes to HCV replication, we next wished to determine whether it interacts with HCV RNA. Cytoplasmic lysate from replicon cells was immunoprecipitated with antibodies to endogenous eIF4AI, eIF4AII or an isotype control. qPCR analysis of immunoprecipitated RNA showed that HCV replicon RNA was immunoprecipitated by the eIF4AII but not eIF4AI or control antibodies (Figure 2A). Given the previously published role for eIF4AII in miRNA function (16), we also tested for the presence of miR-122 in the eIF4AII immunoprecipitate. Similar to HCV RNA, miR-122 was found in the eIF4AII but not eIF4AI immunoprecipitate (Figure 2B). Western blot analysis showed that both proteins were specifically immunoprecipitated by their respective antibodies (Figure 2C), although immunoprecipitation of eIF4AI was less efficient. siRNA-mediated depletion of eIF4AII resulted in loss of both HCV RNA (Supplementary Figure S2A and B) and miR-122 (Supplementary Figure S2C and D) from the eIF4AII immunoprecipitate, confirming the specificity of the immunoprecipitation reaction.

### miR-122 is required for eIF4AII–HCV RNA interaction

**Figure 3.**
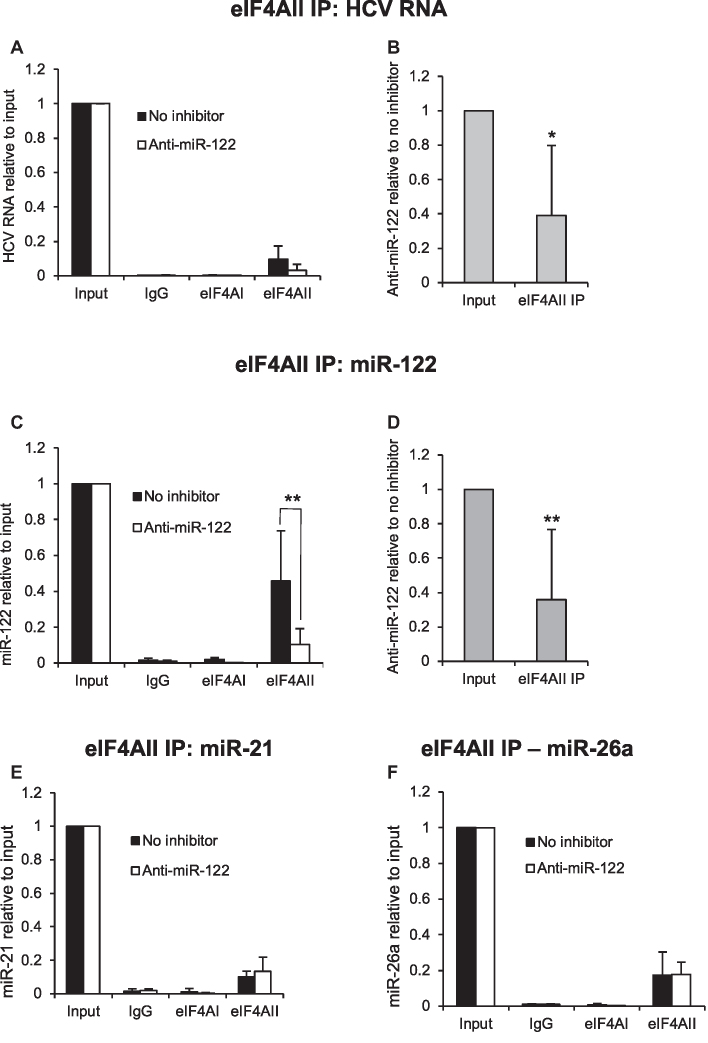
eIF4AII interaction with HCV is dependent on functional miR-122. NNeo/C-5B replicon cells were treated with a miR-122 inhibitor for 6 h prior to immunoprecipitation of cytoplasmic lysate with antibodies specific to eIF4AI, eIF4AII, or an IgG control. (**A**) HCV RNA levels in the immunoprecipitates were measured by qPCR and are shown relative to 25% input. Mean of six independent experiments + SD. (**B**) The data in (A) are shown as the ratio of anti-miR-122:no inhibitor for each experiment in eIF4AII IP relative to input. This ratio was reduced in the eIF4AII IP. *P = 0.029, one sample Student's *t* test. (**C**) As (A), except that miR-122 levels were measured by qPCR. Mean of seven independent experiments + SD. **P = 0.0079, Student's *t* test. (**D**) miR-122 data from (C) were analyzed as in (B). Anti-miR-122:no inhibitor ratio was reduced in eIF4AII IP compared to input. **P = 0.006, one sample Student's *t* test. (**E**) As (A), except that miR-21 levels were measured by qPCR. Mean of four independent experiments + SD. (**F**) As (A), except that miR-26a levels were analyzed by qPCR. Mean of five independent experiments + SD.

The association of eIF4AII with both HCV RNA and miR-122 suggested that miR-122 could contribute to the eIF4AII–HCV RNA interaction. To examine this possibility, replicon cells were subjected to a short (6 h) treatment with a miR-122 inhibitor (anti-miR-122) prior to eIF4AII immunoprecipitation. We observed a reduction in the proportion of HCV replicon RNA associated with eIF4AII (Figure 3A). Despite some variability in the efficiency of immunoprecipitation leading to high experimental error, the ratio of HCV RNA in anti-miR-122:control treatment was significantly lower in eIF4AII IP than in the input lysate, showing a 60% reduction (Figure 3B). This suggests that eIF4AII may be recruited to HCV RNA via miR-122. We also observed a significant 64% reduction in miR-122 in the eIF4AII immunoprecipitate compared to input following transfection of the miR-122 inhibitor (Figure 3C and D). This indicates that the eIF4AII interaction with miR-122 is reduced when miR-122 function is inhibited. Finally, we found that while eIF4AII also associates with two other miRNAs that are highly expressed in the liver, miR-21 and miR-26a, this interaction was not affected by miR-122 inhibition (Figure 3E and F). This demonstrates for the first time that eIF4AII, which was previously shown to regulate miRNA function (16), also interacts with several tested miRNAs. It also indicates that the requirement for functional miR-122 is specific to the eIF4AII interactions with miR-122 and HCV RNA.

### eIF4AI depletion relieves effects of eIF4AII knockdown on HCV RNA levels

**Figure 4.**
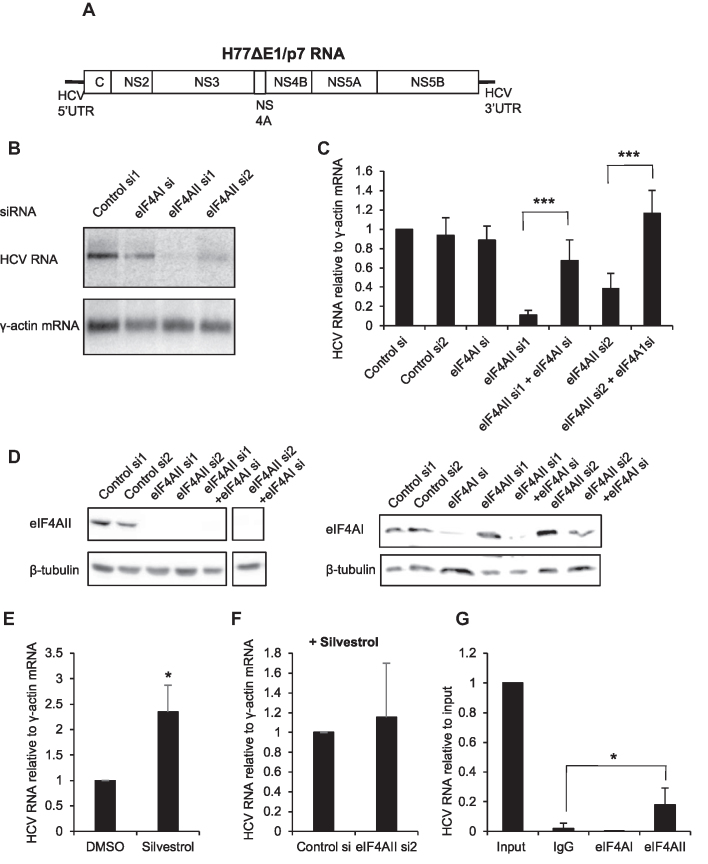
eIF4AI and eIF4AII interact to regulate replication of genotype 1a monocistronic HCV. (**A**) Structure of the H77ΔE1/p7 RNA. (**B**) Northern blot showing HCV RNA in Huh7 cells electroporated with H77ΔE1/p7 RNA, then treated with a non-targeting control siRNA, an siRNA specific to eIF4AI, or one of two siRNAs specific to eIF4AII. γ-actin mRNA is shown as a loading control. HCV RNA levels were reduced by both eIF4AII siRNAs. (**C**) Huh7 cells electroporated with H77ΔE1/p7 RNA were transfected with the two eIF4AII siRNAs, with or without inclusion of the eIF4AI siRNA. HCV RNA relative to a γ-actin mRNA control was measured by qPCR. eIF4AI knockdown relieved the inhibitory effects of eIF4AII knockdown on HCV RNA levels. A second control siRNA was also included in this experiment. Mean of at least three independent experiments + SD. ***P = 0.00047 for eIF4AII si1+eIF4AI si relative to eIF4AII si1 alone, *P* = 0.00011 for eIF4AII si2 + eIF4AI relative to eIF4AII si2 alone, Student's *t* test. (**D**) Western blot showing effective knockdown of eIF4AII by both eIF4AII si1 and si2, with and without inclusion of eIF4AI si, and of eIF4AI with and without inclusion of eIF4AII siRNAs. β-Tubulin is shown as a loading control. (**E**) HCV RNA relative to a γ-actin mRNA control increased when cells electroporated with H77ΔE1/p7 RNA were treated with silvestrol. *P = 0.047. Mean of three independent experiments + SD. (**F**) eIF4AII depletion did not affect HCV RNA levels in cells electroporated with H77ΔE1/p7 RNA and treated with silvestrol. Mean of three independent experiments + SD. (**G**) Cytoplasmic lysate from Huh7 cells electroporated with H77ΔE1/p7 RNA was immunoprecipitated with antibodies to eIF4AI, eIF4AII or an IgG control. HCV RNA levels were determined by qPCR relative to 25% input. Mean of four independent experiments + SD. *P = 0.035, Student's *t* test.

To further investigate the regulation of HCV by eIF4AII in a biochemically amenable system, the knockdown experiments were repeated in Huh7 cells electroporated with a monocistronic, genotype 1a H77ΔE1/p7 RNA (Figure 4A). HCV RNA levels were analyzed by northern blot and showed a decrease following transfection with both eIF4AII siRNAs, although the effect was larger for eIF4AII si1 (Figure 4B). This confirms that eIF4AII is required for effective HCV replication. A decrease in HCV RNA following transfection of both eIF4AII siRNAs into H77ΔE1/p7 electroporated cells was also observed by qPCR, which showed that HCV RNA levels were unaffected by a second control siRNA, or by eIF4AI knockdown (Figure 4C). We also tested the effects of transfecting cells with eIF4AI and eIF4AII siRNA together. Interestingly, we found that eIF4AI depletion relieved the inhibitory effects of both eIF4AII siRNAs on HCV RNA levels (Figure 4C). Western blotting confirmed effective knockdown of eIF4AII by eIF4AII si2 and showed that eIF4AI and eIF4AII siRNAs were both effective in the double knockdowns (Figure 4D). To further investigate the role of eIF4AI in HCV replication, cells electroporated with H77ΔE1/p7 RNA were treated with the eIF4A inhibitor silvestrol. Silvestrol is a member of the rocaglate drug family, which act by causing eIF4A to clamp onto target RNA rather than functioning as a helicase (25). Interestingly, silvestrol treatment led to an increase in HCV RNA levels (Figure 4E) and eIF4AII depletion no longer affected HCV RNA levels when cells were treated with silvestrol (Figure 4F). Together, these data indicate that eIF4AII is only required for HCV replication in the presence of eIF4AI helicase activity.

H77ΔE1/p7 RNA was immunoprecipitated with an antibody to eIF4AII, but not eIF4AI (Figure 4G), confirming the eIF4AII–HCV RNA interaction shown in Figure 2 is shared by genotypes 1a and 1b and is not mediated by the EMCV IRES in the replicon RNA. We also confirmed that miR-122 was immunoprecipitated by the eIF4AII, but not eIF4AI, antibody in Huh7 cells electroporated with H77ΔE1/p7 RNA (Supplementary Figure S3).

### eIF4AII contributes to miR-122 regulation of HCV IRES-driven translation

**Figure 5.**
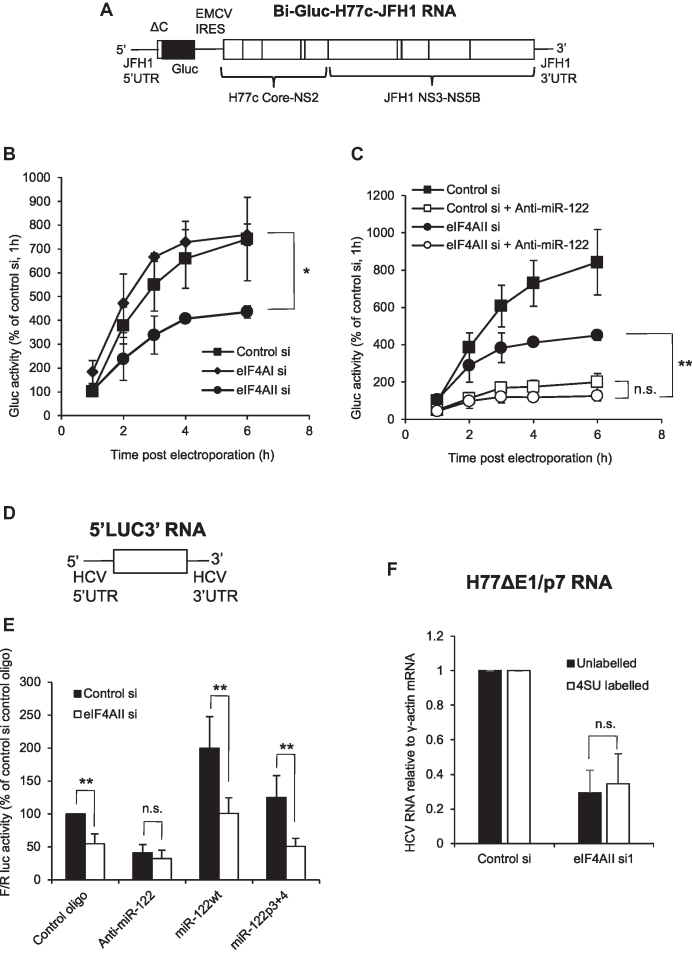
eIF4AII contributes to HCV IRES-driven translation. (**A**) Structure of the Bi-Gluc-H77-JFH1 RNA, in which the HCV 5′UTR drives synthesis of *Gaussia* luciferase. (**B**) Huh7.5 cells treated with siRNA specific to eIF4AI, eIF4AII or a non-targeting control were electroporated with Bi-Gluc-H77-JFH1 RNA. Secreted Gluc was measured in the cell supernatant over a timecourse following electroporation and is shown relative to the level in control siRNA-treated cells at 1 h post-electroporation. Mean of three independent experiments + SD. *P = 0.019 for eIF4AII si compared to control si, Student's *t* test. (**C**) As (B), except that cells treated with eIF4AII siRNA or control siRNA were electroporated with Bi-Gluc-H77-JFH1 RNA with or without a miR-122 inhibitor (anti-miR-122). Mean of three independent experiments + SD. **P = 0.005 for eIF4AII si + anti-miR-122 compared to eIF4AII si alone, Student's *t* test. (**D**) Structure of the 5′LUC3′ reporter RNA. (**E**) Huh7 cells treated with eIF4AII siRNA or control siRNA were transfected with 5′LUC3′ RNA, in combination with a miR-122 inhibitor (Anti-miR-122), a randomized control (Control oligo), wildtype miR-122 (miR122wt) or a mutant non-targeting control (miR122p3+4). Firefly luciferase was measured relative to a *Renilla* luciferase transfection control at 6 h post-transfection and is shown relative to control siRNA, control oligo treatment. Mean of four independent experiments + SD. For eIF4AII si compared to control si, **P = 0.0092 for control oligo, one sample Student's *t* test. **P = 0.0096 for miR122wt, **P = 0.0055 for miR122p3+4, Student's *t* test. (**F**) Cells electroporated with H77ΔE1/p7 RNA were treated with eIF4AII si1 or a non-targeting control for 48 h and labeled with 4SU for 1 h. HCV RNA relative to γ-actin mRNA levels were determined by qPCR and were similarly affected by eIF4AII knockdown in unlabeled and labeled RNA fractions. Mean of four independent experiments + SD.

To dissect the stage of the HCV replication cycle that is regulated by eIF4AII, we tested whether HCV IRES-driven translation is affected. Huh7 cells were electroporated with a bicistronic infectious virus in which the HCV 5′UTR controls production of *Gaussia* luciferase (Gluc) (Figure 5A). Gluc is secreted and can be measured directly from the culture medium. When measured at early times following introduction of the viral RNA before replication occurs, this reflects translation of the input RNA. When cells were transfected with eIF4AII siRNA before electroporation, there was a strong reduction in Gluc production. This was observable as early as 2 h post-electroporation, suggesting that eIF4AII directly modulates HCV translation (Figure 5B). eIF4AI depletion did not affect Gluc production.

We then used the same system to test whether eIF4AII regulates HCV translation via miR-122. Cells transfected with eIF4AII or control siRNAs were electroporated with the Gluc replicon RNA with or without a miR-122 inhibitor (anti-miR-122). miR-122 inhibition strongly decreased early translation mediated by the HCV IRES (Figure 5C). Following eIF4AII knockdown, the miR-122 inhibitor also decreased Gluc production at early timepoints, indicating that endogenous miR-122 contributes to HCV translation under conditions of eIF4AII depletion (Figure 5C). However, under conditions of miR-122 inhibition, there was no significant effect of eIF4AII knockdown. This suggests that eIF4AII only stimulates HCV translation in the presence of endogenous miR-122. Coupled with our observation that the eIF4AII–HCV RNA interaction requires functional miR-122 (Figure 3A and B), this suggests that miR-122-dependent recruitment might be necessary for eIF4AII to regulate HCV translation.

To further investigate the role for eIF4AII in regulation of HCV translation, we used a reporter system we previously developed to test the effects of miR-122 on HCV 5′UTR driven translation. This reporter consists of the firefly luciferase coding region flanked by the full HCV 5′ and 3′UTRs, which is delivered into cells as an uncapped RNA (Figure 5D) (9). Inclusion of a miR-122 inhibitor (anti-miR-122) led to the expected decrease in luciferase activity in control siRNA-treated cells (Figure 5E), while miR-122 overexpression (miR122wt) led to an increase in firefly luciferase, confirming that miR-122 stimulates translation driven by the HCV 5′UTR. A randomized 2′OMe oligonucleotide (control oligo) served as a control for the miR-122 inhibitor, while miR-122 with mutations in its seed (miR122p3+4) acted as a control for miR-122 overexpression. When this experiment was carried out in cells depleted of eIF4AII, a decrease in firefly luciferase was seen under control conditions (control oligo and miR122p3+4), confirming that eIF4AII contributes to HCV IRES-driven translation (Figure 5E). Conversely, eIF4AII knockdown did not affect luciferase expression under conditions of miR-122 inhibition (Anti-miR-122). Taken together, these results suggest that the eIF4AII effect on HCV IRES-driven translation is mediated at least partially by miR-122.

To assess whether the inhibitory effect of eIF4AII depletion on HCV 5′UTR-driven translation could be alleviated by eIF4AI knockdown, as seen for HCV replication in Figure 4C, combined knockdown of eIF4AI and eIF4AII was carried out before transfection of Huh7 cells with 5′LUC3′ RNA with control oligo or anti-miR-122. The inhibitory effect of eIF4AII knockdown on HCV 5′UTR-driven translation was lost (Supplementary Figure S4A), although this could be partly attributable to reduced translation of the cap-dependent *Renilla* luciferase control under conditions of eIF4AI knockdown. We also tested whether eIF4AII affects translation mediated by the HCV IRES in the context of a bicistronic reporter plasmid (Supplementary Figure S4B) (26). While eIF4AI knockdown increased the firefly/*Renilla* luciferase ratio, due to inhibition of cap-dependent *Renilla* luciferase translation, eIF4AII knockdown had no effect on the ratio (Supplementary Figure S4C). As eIF4AII knockdown does not affect cap-dependent translation (data not shown, (17)), this indicates that HCV IRES-dependent translation was also unaffected. The HCV IRES in the bicistronic plasmid lacks the miR-122 binding region, suggesting that eIF4AII only contributes to HCV IRES-driven translation in the context of miR-122 regulation.

The role for miR-122 in HCV 5′UTR-driven translation does not exclude potential additional roles in other stages of the HCV replication cycle. To directly assess whether eIF4AII depletion affects nascent HCV RNA synthesis, we carried out 4-thio-uridine (4SU) labeling of Huh7 cells electroporated with H77ΔE1/p7 RNA and treated with eIF4AII siRNA or a non-targeting control. This approach allows RNA synthesized during the 4SU treatment period to be isolated by biotinylation and strepatavidin purification (22,23). Following 1h 4SU treatment, we observed an equivalent decrease in HCV RNA in both the unlabeled and labeled fractions (Figure 5F). The decrease in the labeled fraction is in accord with the reduced availability of template in the unlabeled fraction and indicates that HCV RNA synthesis is not directly affected by eIF4AII knockdown, as has also been shown for miR-122 inhibition (27). However, it does not exclude the possibility that eIF4AII regulates other stages of the HCV replication cycle that are difficult to analyze directly, such as the translation-replication switch, in which miR-122 has been implicated (15).

### eIF4AII contributes to miR-122 regulation of HCV replication

**Figure 6.**
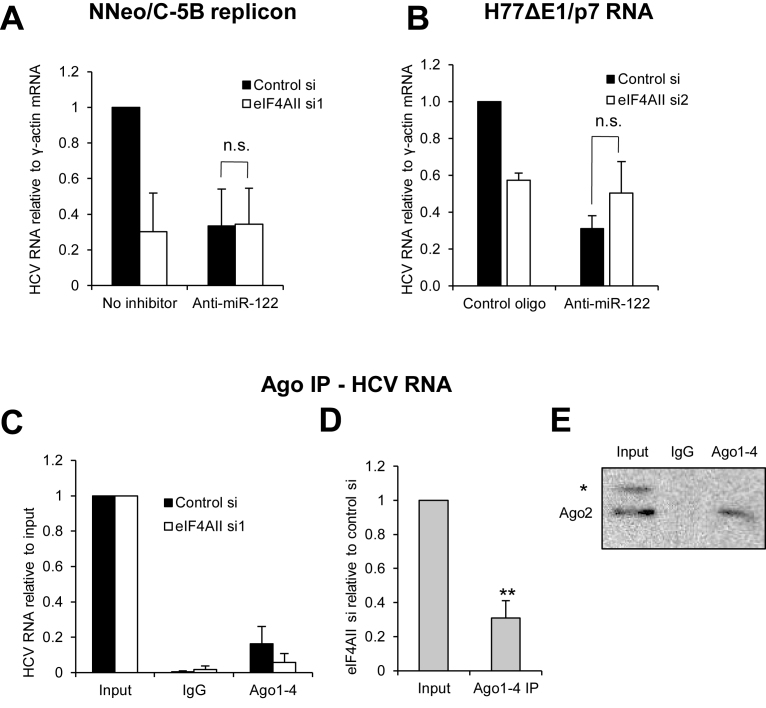
The miRNA machinery is involved in eIF4AII regulation of HCV replication. (**A**) NNeo/C-5B replicon cells were treated with siRNA to eIF4AII or a non-targeting control, with or without inclusion of a miR-122 inhibitor (Anti-miR-122). HCV RNA levels were determined by qPCR relative to γ-actin mRNA at 72 h. There was no significant effect of eIF4AII knockdown in the presence of the miR-122 inhibitor. Mean of 3 independent experiments + SD. (**B**) Huh7 cells electroporated with monocistronic H77ΔE1/p7 RNA were treated with eIF4AII si2 or control si for 48 h, then transfected with miR-122 inhibitor or a control oligo for 24 h. Total RNA was harvested and HCV RNA levels were determined by qPCR relative to γ-actin mRNA. Mean of three independent experiments + SD. (**C**) The RISC was immunoprecipitated with an antibody to Ago1–4 in Huh7 cells electroporated with monocistronic H77ΔE1/p7 RNA and treated with eIF4AII siRNA or a non-targeting control. HCV RNA levels in control IgG or Ago immunoprecipitates were determined relative to 25% input by qPCR. Data show mean of three independent experiments + SD. (**D**) The data in (C) are shown as a mean of the ratio of HCV RNA levels in eIF4AII si:control si for each experiment in Ago IP relative to input. **P = 0.007, one sample Student's *t* test. (**E**) Protein was extracted from IgG and Ago1–4 immunoprecipitates and 25% input and subjected to western blotting with an antibody to Ago2, which shows that pulldown was specific. * indicates a non-specific band.

The data above suggest that eIF4AII regulates HCV translation, at least in part, by modulation of miR-122 regulation. To investigate whether this also applies in the context of viral replication, a miR-122 inhibitor (anti-miR-122) and eIF4AII siRNA were introduced into replicon cells. qPCR analysis showed a decrease in HCV RNA levels when a miR-122 inhibitor was present in control siRNA-treated cells (Figure 6A), as previously shown (5). However, anti-miR-122 did not affect HCV RNA levels in eIF4AII knockdown cells, supporting the idea that eIF4AII contributes to miR-122 regulation of HCV replication. A similar experiment was then carried out in Huh7 cells electroporated with H77ΔE1/p7 RNA, except that in this case the miR-122 inhibitor or a control oligo was transfected into cells 48 h after siRNA transfection, to avoid any potential effects of oligonucleotide inclusion on siRNA transfection efficiency. Again, miR-122 inhibition led to a decrease in HCV RNA in control siRNA-treated cells but not in eIF4AII knockdown cells (Figure 6B). We also tested the effects of eIF4AII depletion on replication of a miR-122-independent HCV RNA, in which a segment of the U3 snoRNA replaces the miR-122 binding sites (28). In both wildtype Huh7.5 and Δ122 mutant cells, in which miR-122 is deleted by CRISPR (29), eIF4AII knockdown had no effect on HCV RNA levels (Supplementary Figure S5).

Next, we immunoprecipitated cytoplasmic lysates of cells containing H77ΔE1/p7 RNA with an antibody that recognizes all four human Ago proteins. Both our group and others have previously shown that Ago association with HCV RNA is largely mediated via miR-122 binding (21,29). qPCR was used to measure the amount of HCV RNA in Ago1–4 and control IgG immunoprecipitates relative to 25% input. We found that eIF4AII depletion reduced the association of Ago with HCV RNA (Figure 6C). When the ratio of HCV RNA in the Ago immunoprecipitate versus input was determined in eIF4AII knockdown cells relative to control siRNA treated cells, we observed a statistically significant 69% decrease (Figure 6D). Western blotting confirmed the Ago IP was effective (Figure 6E). eIF4AII knockdown did not affect the level of miR-122 (Supplementary Figure S6A) or its association with Ago1–4 (Supplementary Figure S6B and C), suggesting that eIF4AII specifically contributes to miR-122 recruitment to, or retention on, HCV RNA.

We also wished to determine whether eIF4AI knockdown could relieve the inhibition of HCV translation and replication by a miR-122 inhibitor. Using both the Gluc assay as a measure of translation (Supplementary Figure S7A) and quantification of HCV RNA levels in cells electroporated with H77ΔE1/p7 RNA (Supplementary Figure S7B), we did not observe any relief of the inhibitory effects of anti-miR-122 when cells were transfected with an eIF4AI siRNA. Together, these data suggest that eIF4AII is only required for HCV replication when eIF4AI is functional, probably due to a role in preventing miR-122 displacement by eIF4AI, but that miR-122 regulation of HCV lies downstream of eIF4AI/eIF4AII and is thus unaffected by eIF4AI knockdown.

### Interplay between CNOT1 and eIF4A proteins in HCV replication

**Figure 7.**
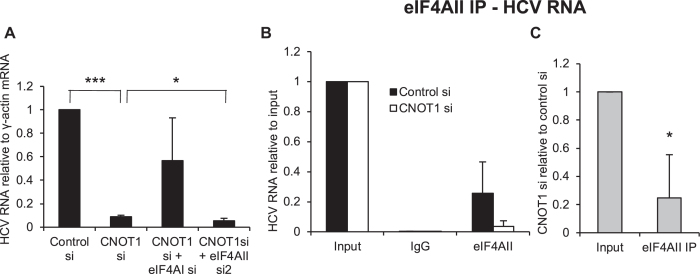
Regulation of HCV replication by eIF4AII and miR-122 is affected by CNOT1. (A) Huh7 cells electroporated with H77ΔE1/p7 RNA were transfected with CNOT1 siRNA, with or without inclusion of the eIF4AI or eIF4AII siRNA. HCV RNA relative to a γ-actin mRNA control was measured by qPCR at 72 h post-transfection. Mean of four independent experiments + SD. ***P < 0.0001, one sample Student's *t* test. *P = 0.036, Student's *t* test. (**B**) NNeo/C-5B replicon cells were transfected with an siRNA specific to CNOT1 or a non-targeting control siRNA for 72 h before immunoprecipitation with an eIF4AII antibody or an IgG control. RNA was extracted from immunoprecipitates and HCV RNA levels determined by qPCR. Mean of three independent experiments + SD. (**C**) The data in (B) are shown as the mean ratio of CNOT1 si:control si for each experiment in eIF4AII IP relative to input, + SD. *P = 0.016, one sample Student's *t* test.

The protein CNOT1 is a central component of the CCR4-NOT deadenylase complex and has been previously identified as a host factor for HCV (30–32). CNOT1 also functions in miRNA activity at 3′UTR sites, as it is recruited to miRNA 3′UTR targets through interaction with the RISC component TNRC6A-C, leading to mRNA deadenylation and decay and to translational repression (33–36). An interaction between the CCR4-NOT complex and eIF4AII has also been identified (16). To investigate whether CNOT1 is involved in eIF4AII regulation of HCV, we transfected Huh7 cells containing the H77ΔE1/p7 replicating RNA with a CNOT1 siRNA and confirmed that this strongly decreases HCV RNA levels (Figure 7A). Interestingly, we found that inclusion of eIF4AI siRNA relieved the effect of CNOT1 knockdown on HCV RNA levels (Figure 7A). Although experimental variability meant that the effect was not quite significant (*P* = 0.056), the effect size was large (fivefold increase). This is similar to our observations with eIF4AII knockdown (Figure 4C), suggesting that at least part of the CNOT1 function in HCV replication may be antagonistic to eIF4AI and that eIF4AII and CNOT1 may regulate HCV through a common pathway. In contrast, depletion of eIF4AII and CNOT1 together led to a statistically significant further decrease in HCV RNA levels compared to depletion of CNOT1 alone, although the effect size was small (Figure 7A). We also found that depletion of CNOT1 reduced the immunoprecipitation of HCV RNA by an eIF4AII antibody (Figure 7B). When the ratio of CNOT1 si:control si in eIF4AII IP was compared to that in input RNA, a significant 75% decrease was observed (Figure 7C). This suggests that eIF4AII is recruited to HCV RNA by CNOT1 and supports the idea that eIF4AII and CNOT1 regulate HCV via a common pathway.

## DISCUSSION

Here, we identify eIF4AII as a host factor that supports HCV replication. We show that eIF4AII contributes to HCV replication in several different viral replication systems with different genotypes. Our data indicate that eIF4AII regulation of HCV is modulated at least in part via miR-122. Interestingly, we find that the closely related protein eIF4AI does not exert the same effects on HCV, and indeed that eIF4AI depletion or inhibition with silvestrol relieves the inhibitory effects of eIF4AII knockdown on HCV replication, suggesting antagonistic functions for eIF4AI and eIF4AII.

A role for eIF4AII in miRNA-mediated translation repression was previously identified (16). However, this is controversial, as eIF4AII did not affect the silencing of mRNAs by tethered RISC or NOT1 (36) and CRISPR/Cas9-mediated knockout of eIF4AII did not affect miRNA-mediated repression (37). CNOT1 is known to be involved in miRNA-mediated repression at 3′UTR sites, where it functions to bring the CCR4-NOT deadenylase complex to the targeted RNA (33–35), and the CCR4-NOT complex was shown to interact with eIF4AII (16,38). However, other studies showed instead that DDX6 is the factor that is recruited by the RISC at miRNA-targeted 3′UTRs and both interacts with CNOT1 and mediates translational repression (36,39–40). It is possible that these processes are dynamic and either eIF4AII or DDX6 may be involved, depending on the cell type, physiological state, or the miRNA and target mRNA involved. It is also possible that cells selected for stable knockout of eIF4AII have developed compensatory mechanisms.

Interestingly, DDX6 is also a host factor for HCV replication, although the mechanism underlying this is not clear. DDX6 was shown to affect HCV translation in some studies (41,42), but not in others (43,44). CNOT1 was also previously identified as a host factor for HCV (30–32). eIF4AII therefore joins DDX6 and CNOT1 in a growing list of proteins that contribute to HCV replication but have also been implicated in miRNA-mediated repression. However, it has been difficult to demonstrate conclusively whether any of these proteins regulate HCV via miR-122, as this is challenging to test experimentally due to the complexity of the HCV replication cycle and the difficulties in drawing firm conclusions from knockdown experiments. DDX6 regulation of HCV was shown to be independent of miR-122 (42,43), but in a recent publication DDX6 was shown to contribute to miR-122 regulation of HCV via the second of its two 5′UTR binding sites (44).

In this study, we provide several lines of evidence that support the idea that at least part of the eIF4AII regulation of HCV occurs via modulation of miR-122 activity. First, we show that the eIF4AII–HCV RNA interaction is miR-122-dependent (Figure 3A and B), suggesting that eIF4AII is recruited to HCV RNA via miR-122. As we also observed a decrease in eIF4AII–HCV RNA interaction following CNOT1 depletion (Figure 7B and C), it is likely that eIF4AII is recruited via the miR-122-RISC interaction with CNOT1 although further work will be necessary to confirm exactly how CNOT1 and eIF4AII interact in the regulation of HCV. Secondly, the association between Ago and HCV RNA, which is largely mediated via miR-122 (29), was reduced when eIF4AII was depleted, suggesting a role for eIF4AII in miR-122-RISC binding to, or retention on, HCV RNA (Figure 6C and D). Finally, we found that the extent of inhibition of HCV IRES-driven translation or HCV replication by eIF4AII depletion was reduced under conditions of miR-122 inhibition (Figures 5 and 6). Further work will be necessary to determine exactly how these factors interact on HCV RNA and to characterize the relationship between eIF4AII and other proteins involved in miR-122 regulation of HCV, such as DDX6 (44), Xrn1/2 (11–13) and PCBP2 (15).

Interestingly, our results indicate that eIF4AII is only required for HCV replication in the presence of functional eIF4AI (Figure 4C–F) and suggest that this is also true of CNOT1 (Figure 7A). Together with our observation that eIF4AII interacts with HCV RNA, this leads us to propose a model in which eIF4AI and eIF4AII compete for interaction with HCV RNA. When eIF4AII is depleted, eIF4AI can interact and inhibit HCV replication. eIF4AII depletion reduces Ago–HCV RNA interaction (Figure 6C and D), supporting the idea that when eIF4AII is absent eIF4AI is recruited and displaces miR-122 from its binding sites on HCV. Silvestrol treatment, which causes eIF4AI to bind to target mRNAs as a clamp (25), increases HCV replication and alleviates the inhibitory effects of eIF4AII depletion (Figure 4E and F). We believe the most likely explanation for our data is a model in which the helicase activity of eIF4AI can displace the miR-122-RISC complex from the HCV 5′UTR, but eIF4AII (probably binding in association with CNOT1) competes with eIF4AI for binding and allows miR-122 to bind to and activate translation/replication of HCV RNA. eIF4AII depletion allows eIF4AI to displace miR-122, resulting in reduced association of Ago with HCV RNA, and reduced HCV RNA levels and translation due to a reduction in miR-122 association and therefore regulation. Such displacement would explain why inclusion of anti-miR-122 in the eIF4AII knockdown experiments does not lead to further reduction in HCV translation (Figure 5C and E) or replication (Figure 6A and B). Further investigation will be necessary to confirm this model. A previous indication of different functions for eIF4AI and eIF4AII came from knockdown experiments showing that eIF4AI, but not eIF4AII, is required for cell growth (17). The basis for these differences has not been identified, but it is possible that different interactions with cofactors such as CNOT1 or eIF4G may be involved.

Our data show that eIF4AII depletion inhibits HCV IRES-driven translation (Figure 5C and E), but does not affect HCV RNA synthesis (Figure 5F). This is reminiscent of previous observations regarding the mechanism of miR-122 and does not exclude potential roles for eIF4AII at other stages of the HCV replication cycle, such as the translation-replication switch. The exact mechanism of HCV regulation by miR-122 remains uncertain although current data suggest that it may act at multiple stages in the replication cycle. Our data suggest that eIF4AII acts upstream of miR-122 in the regulation of HCV, affecting miR-122 recruitment to, or retention at, HCV RNA but not its downstream regulatory function. This is supported by our observation that the inhibitory effects of eIF4AII knockdown on HCV are relieved by eIF4AI knockdown (Figure 4), but those of miR-122 inhibition are not (Supplementary Figure S7).

Finally, an analogous role for miRNAs in viral replication to that of miR-122 was recently identified for miR-17 and let-7, which bind to bovine viral diarrhoea virus (BVDV) RNA and stimulate viral replication (45). In contrast to miR-122 binding to the HCV 5′UTR, these interactions occur via the 3′ UTR. It would be very interesting to determine whether common host factors are involved in both regulatory processes.

## Supplementary Material

Supplementary Data
