# Supplementary material for: Eukaryotic translation initiation factor 4AII contributes to microRNA-122 regulation of hepatitis C virus replication
Source: Nucleic Acids Res. 2018 Apr 14;46(12):6330–43. doi: 10.1093/nar/gky262 (PMC6158612; doi:10.1093/nar/gky262)
Supplement: Supplementary Data [file gky262_supplemental_figures.pdf]

Figure S1

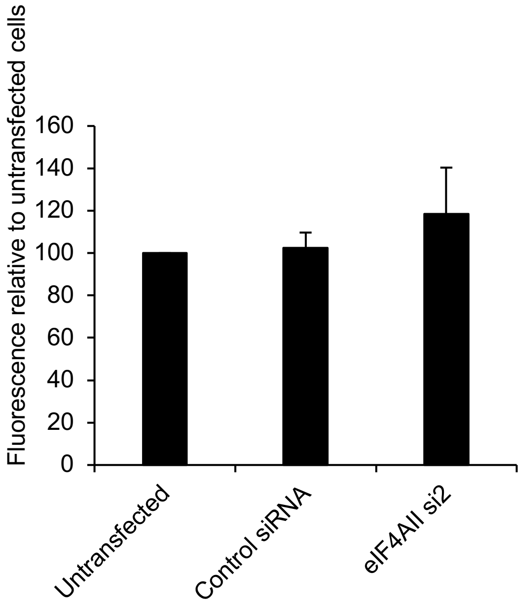

Figure S1. eIF4AII knockdown does not affect cell viability. Huh7 cells were transfected with eIF4AII si2 or a non-targeting control for 72h before treatment with AlamarBlue for 3h. Fluorescence was quantified and is shown relative to an untransfected control. Mean of 3 independent experiments +SD.

Figure S2

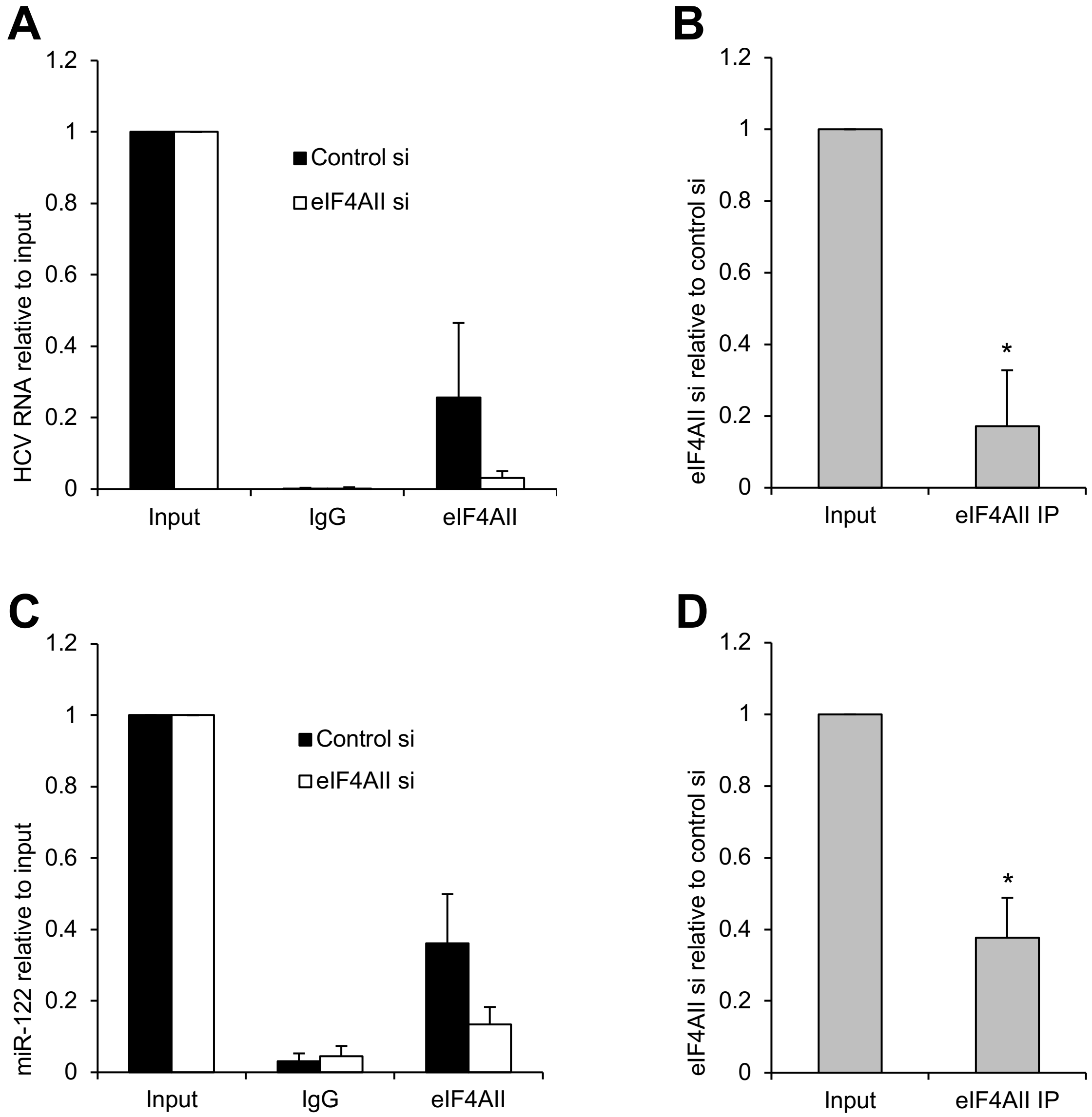

Figure S2. eIF4AII immunoprecipitation is specific. NNeo/C-5B replicon cells were transfected with an siRNA specific to eIF4AII or a non-targeting control siRNA for 72h before immunoprecipitation with antibodies to eIF4AI, eIF4AII or an IgG control. (A) RNA was extracted from immunoprecipitates and analyzed by qPCR with primers specific to HCV RNA. Mean of 3 independent experiments +SD. (B) The data in (A) were plotted as a mean of the ratio of the amount of HCV RNA in eIF4AII relative to control siRNA-treated cells, +SD. The amount of HCV RNA in the eIF4AII IP in eIF4AII knockdown versus control siRNA-treated cells was significantly lower than in the input. *P=0.012, one sample Student's t test. (C) As (A), except that miR-122 was measured by qPCR. (D) As (B), except miR-122 data are shown. The amount of miR-122 in the eIF4AII IP in eIF4AII knockdown versus control siRNA-treated cells was significantly lower than in the input. *P=0.011, one sample Student's t test.

Figure S3

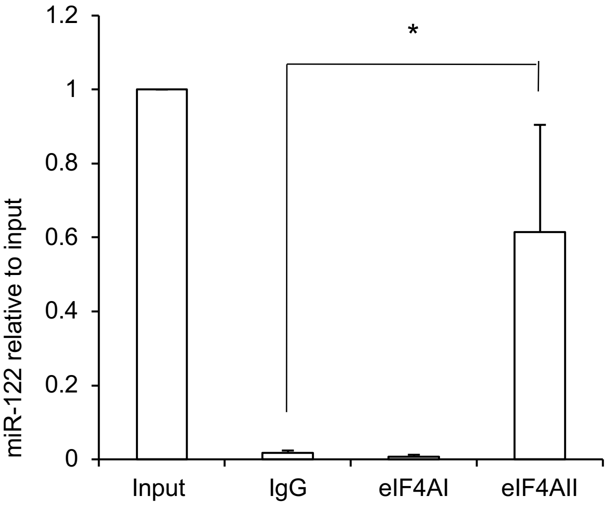

Figure S3. miR-122 associates with endogenous eIF4AII in Huh7 cells electroporated with H77ΔE1/p7 RNA. Cytoplasmic lysate from Huh7 cells electroporated with H77ΔE1/p7 RNA was immunoprecipitated with antibodies to eIF4AI, eIF4AII or an IgG control. miR-122 levels were determined by qPCR relative to 25% input. Mean of 3 independent experiments +SD. *P=0.023, Student's t test.

Figure S4

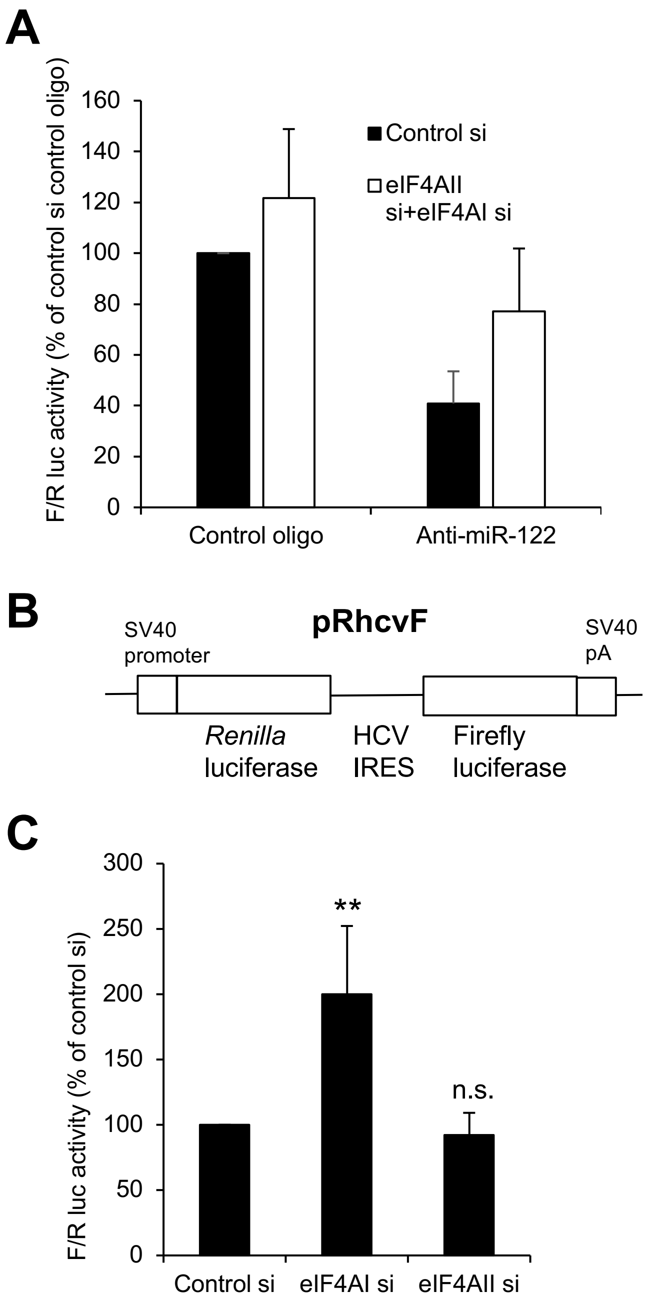

Figure S4. Effects of eIF4AI and eIF4AII knockdown on HCV IRES-driven translation. (A) Huh7 cells were transfected with siRNAs to deplete eIF4AI and eIF4AII in combination, or a non-targeting control, before transfection with 5’LUC3’ RNA as in figure 5E. Firefly luciferase was measured relative to a *Renilla* luciferase transfection control at 6h post transfection and is shown relative to control siRNA, control oligo treatment. Mean of 4 independent experiments, +SD. (B) Schematic diagram showing the pRhcvF bicistronic plasmid. (C) Huh7 cells treated with siRNAs specific to eIF4AI, eIF4AII, or a non-targeting control were transfected with pRhcvF. Firefly/*Renilla* luciferase activity was determined at 24h post plasmid transfection and is shown relative to control si treatment. Mean of 7 independent experiments, +SD. ** P=0.0063, one sample t test.

Figure S5

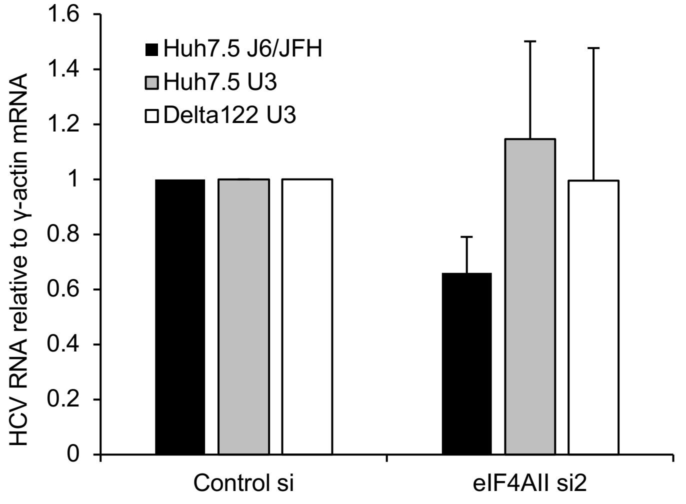

Figure S5. eIF4AII does not affect replication of miR-122-independent HCV. Huh7.5 cells were electroporated with J6/JFH1 RNA, and Huh7.5 and ΔmiR-122 cells electroporated with U3 mutant RNA, for 6h before transfection with eIF4AII si2 or a non-targeting control siRNA. RNA was harvested at 72h post transfection and HCV RNA levels determined relative to a γ-actin mRNA control. Mean of three independent experiments +SD.

Figure S6

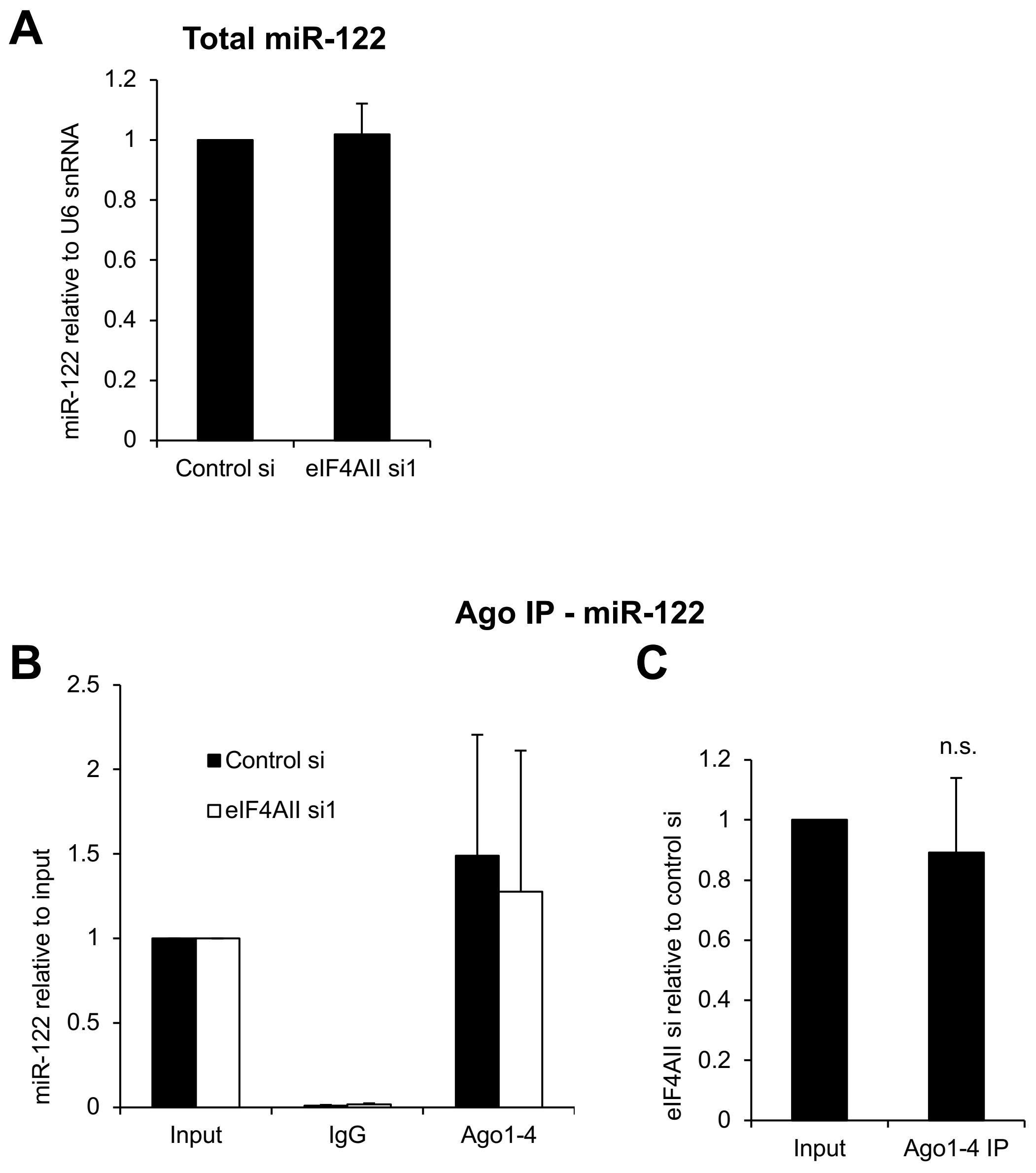

Figure S6. eIF4AII depletion does not affect miR-122 levels or RISC incorporation. (A) miR-122 levels relative to U6 snRNA were measured by qPCR in total RNA extracted from Huh7 cells treated with siRNA to eIF4AII or a non-targeting control. Mean of 3 independent experiments +SD. (B) The RISC was immunoprecipitated with an antibody to Ago1-4 in Huh7 cells electroporated with monocistronic H77ΔE1/p7 RNA and treated with eIF4AII siRNA or a non-targeting control. miR-122 levels in control IgG or Ago1-4 immunoprecipitates were determined relative to 25% input by qPCR. Mean of 4 independent experiments +SD. (C) The data in (B) are shown as a mean of the ratio of miR-122 levels in eIF4AII si:control si for each experiment in Ago IP relative to input.

Figure S7

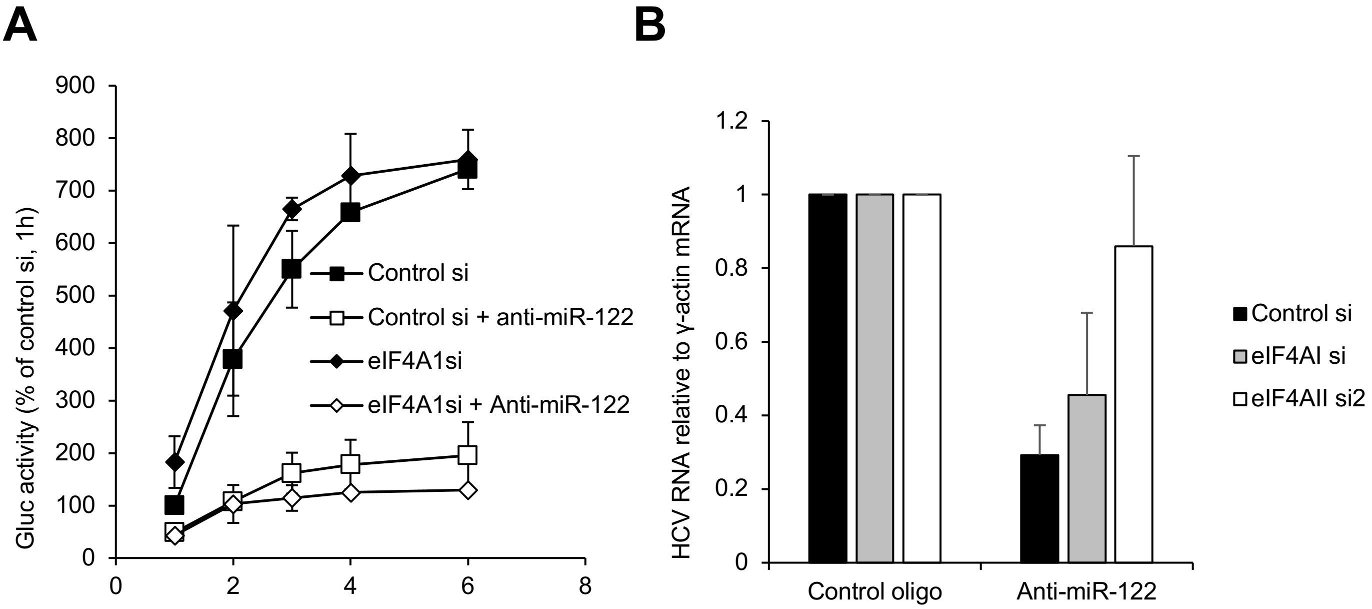

Figure S7. eIF4AI depletion does not relieve the inhibitory effects of miR-122 inhibition on HCV translation and replication. (A) Huh7.5 cells treated with eIF4AI siRNA or control siRNA were electroporated with Bi-Gluc-H77-JFH1 RNA with or without a miR-122 inhibitor (anti-miR-122). Secreted Gluc was measured in the cell supernatant over a timecourse following electroporation and is shown relative to the level in control siRNA-treated cells at 1h post electroporation. Mean of 3 independent experiments +SD. (B) Huh7 cells electroporated with monocistronic H77ΔE1/p7 RNA were treated with eIF4AI si or control si for 48h, then transfected with anti-miR-122 or a control oligo for 24h. Total RNA was harvested and HCV RNA levels were determined by qPCR relative to γ-actin mRNA. Mean of 3 independent experiments +SD.

Supplementary materials and methods

Plasmids and cell lines

The plasmid pRhcvF was a kind gift of Anne Willis (MRC Toxicology Unit, Leicester) (27). The plasmids J6/JFH1 clone 2 and J6/JFH1-U3 and the ΔmiR-122 cell line were kind gifts of Charles Rice, Rockefeller University (30).

AlamarBlue assay

Cell proliferation was measured using the AlamarBlue reagent (ThermoFisher), according to the manufacturer's instructions. Huh7 cells were transfected with eIF4AII or non-targeting control siRNAs in 6cm plates. At 48h post transfection, cells were plated in 96 well plates in quadruplicate, and 24h later were treated with AlamarBlue for 3h. Fluorescence was measured using a Biotek Synergy HT plate reader.

Bicistronic vector transfection

Huh7 cells were transfected with siRNAs in 6 well plates 48h before plasmid transfection. The day before transfection, cells were split into 24 well plates. 0.1μg plasmid was delivered per 3 wells using lipofectamine 2000 and luciferase activity determined at 24h post plasmid transfection, as described in materials and methods of main manuscript.
